# Neuromuscular development of *Aeolidiella stephanieae *Valdéz, 2005 (Mollusca, Gastropoda, Nudibranchia)

**DOI:** 10.1186/1742-9994-7-5

**Published:** 2010-01-22

**Authors:** Alen Kristof, Annette Klussmann-Kolb

**Affiliations:** 1 Research Group for Comparative Zoology, Department of Biology, University of Copenhagen, 2100 Copenhagen Ø, Denmark; 2 Institute for Ecology, Evolution & Diversity, Goethe-University, 60054 Frankfurt am Main, Germany

## Abstract

**Background:**

Studies on the development of the nervous system and the musculature of invertebrates have become more sophisticated and numerous within the last decade and have proven to provide new insights into the evolutionary history of organisms. In order to provide new morphogenetic data on opisthobranch gastropods we investigated the neuromuscular development in the nudibranch *Aeolidiella stephanieae *Valdéz, 2005 using immunocytochemistry as well as F-actin labelling in conjunction with confocal laser scanning microscopy (cLSM).

**Results:**

The ontogenetic development of *Aeolidiella stephanieae *can be subdivided into 8 stages, each recognisable by characteristic morphological and behavioural features as well as specific characters of the nervous system and the muscular system, respectively. The larval nervous system of *A. stephanieae *includes an apical organ, developing central ganglia, and peripheral neurons associated with the velum, foot and posterior, visceral part of the larva. The first serotonergic and FMRFamidergic neural structures appear in the apical organ that exhibits an array of three sensory, flask-shaped and two non-sensory, round neurons, which altogether disappear prior to metamorphosis. The postmetamorphic central nervous system (CNS) becomes concentrated, and the rhinophoral ganglia develop together with the *anlage *of the future rhinophores whereas oral tentacle ganglia are not found. The myogenesis in *A. stephanieae *begins with the larval retractor muscle followed by the accessory larval retractor muscle, the velar or prototroch muscles and the pedal retractors that all together degenerate during metamorphosis, and the adult muscle complex forms *de novo*.

**Conclusions:**

*Aeolidiella stephanieae *comprises features of the larval and postmetamorphic nervous as well as muscular system that represent the ground plan of the Mollusca or even the Trochozoa (e. g. presence of the prototrochal or velar muscle ring). On the one hand, *A. stephanieae *shows some features shared by all nudibranchs like the postmetamorphic condensation of the CNS, the possession of rhinophoral ganglia and the lack of oral tentacle ganglia as well as the *de novo *formation of the adult muscle complex. On the other hand, the structure and arrangement of the serotonergic apical organ is similar to other caenogastropod and opisthobranch gastropods supporting their sister group relationship.

## Background

The development of more sophisticated techniques to study the detailed structures of nervous systems as well as muscular systems has provided new insights into the organization of these character complexes and has yielded so far unknown information to understand the evolutionary history of organisms. Some of these studies focussed on the investigation of serotonergic as well as FMRFamidergic characteristics of the nervous system utilizing immunocytochemistry in conjunction with confocal laser scanning microscopy [1-9]. Although these labellings result in an incomplete picture of the nervous system [10,11], they have shown to provide characters that facilitate the identification of homologous portions of the nervous system across different taxa [12-15], thus being of interest in order to infer phylogenetic hypotheses or reveal insights into evolutionary trends [16-25].

The same holds true for investigations of the development of the muscular system which have been applied with similar techniques to various invertebrate taxa including Mollusca [reviewed in Wanninger [25]].

We investigated the development of the central nervous system and the musculature in the nudibranch *Aeolidiella stephanieae *Valdéz, 2005 in order to gain insights into the structure and evolution of these organ systems.

**Figure 1:**
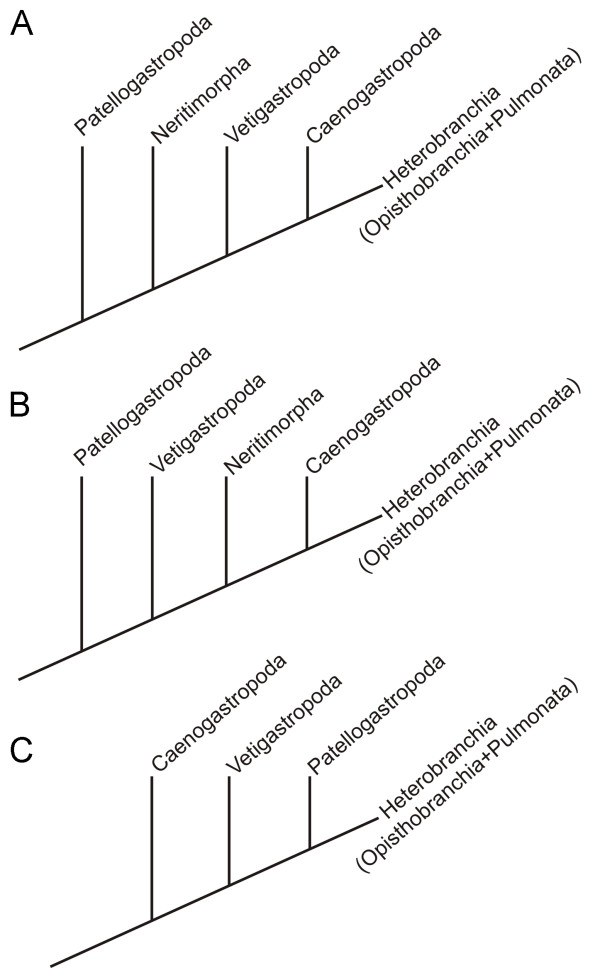
**Phylogenetic relationships among major gastropod lineages**. Note that Heterobranchia additionally to Opisthobranchia and Pulmonata comprises some minor groups (Valvatoidea, Rissoelloidea, Architectonicoidea, and Pyramidelloidea). (A) Morphological phylogenies of Haszprunar [91] and Ponder and Lindberg [47]. (B) Molecular analysis based on genomic DNA by McArthur and Harasewych [37], and combined analysis of molecular and morphological data by Aktipis et al. [38]. (C) Molecular phylogeny based on mitochondrial genome arrangements by Grande et al. [39]; Note that neritimorph gastropods were not included in the analysis.

The Nudibranchia belongs to the Opisthobranchia which represents a morphologically diverse group of gastropods occupying a great variety of ecological niches. Opisthobranchs have a global distribution, but are restricted almost exclusively to marine habitats with the only exception being few freshwater acochlidians [26]. The reduction or loss of the shell, the elaboration of the head, foot or mantle, and the acquisition of chemical defences are evolutionary trends shared by most opisthobranch taxa [27]. In consequence, their phylogenetic history is still not satisfactorily unravelled mainly due to convergent evolution of different character complexes [27,28] and different approaches to infer phylogenetic relationships within Opisthobranchia yield conflicting results [29-35]. Even though many morphological and molecular analyses suggest a paraphyletic Opisthobranchia, the monophyly of the major groups within is well supported, in which Nudibranchia is the most derived lineage [reviewed in Wägele et al. [36]]. Irrespective of whether Opisthobranchia are paraphyletic or not, they are considered as one of the most derived gastropod clades (Fig. 1A-C). However, the relationships among the major molluscan and gastropod lineages have not yet reached agreement [37-40]. Therefore, it is important to investigate additional, phylogenetically informative characters to evaluate present hypotheses on the phylogeny of this taxon. By using immunocytochemical markers for the characterisation of the nervous system and F-actin labelling for muscles of the nudibranch *A. stephanieae *we provide new data that in future studies can help to elucidate phylogenetic or evolutionary relations of these enigmatic animals by comparison with other Opisthobranchia, Gastropoda or Mollusca respectively.

## Results

### Embryogenesis and larval development in *Aeolidiella stephanieae*

**Figure 2:**
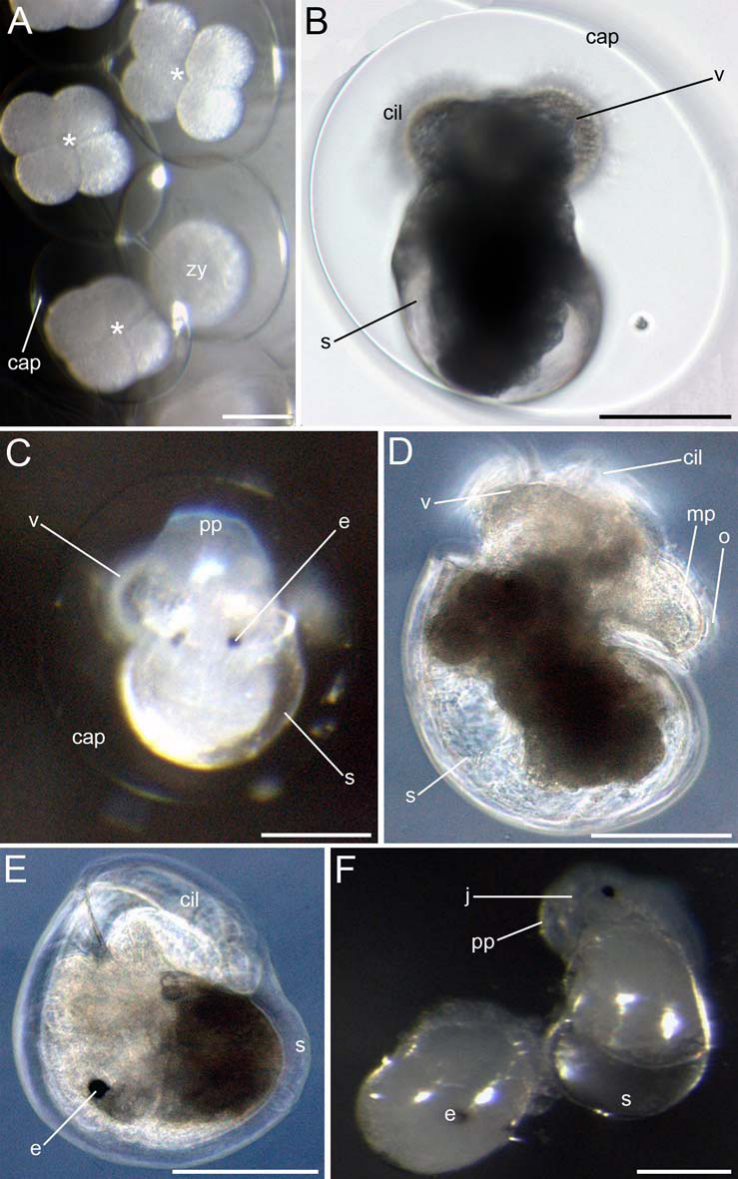
**Embryos and larvae of *Aeolidiella stephanieae***. Light micrographs. Anterior faces upwards and scale bars represent 100 μm in all aspects. B, C and F are in dorsal view whereas D and E are in lateral view. (A) 2 h after oviposition (0% of development); zygotes (zy) and four-celled embryos (asterisk) within the same egg mass; cap marks the capsule that surrounds each embryo. (B) Early veliger stage larva (5% of development) with a shell (s) and a ciliated velum (v). (C) Veliger stage larva (12% of development) with eyes (e) and elaborated larval foot (propodium) (pp). (D) Hatched larva (20% of development) with the operculum (o) that is attached to the metapodium (mp). (E) Retracted and settled larva in metamorphosis (25% of development). (F) Early juvenile stage *Aeolidiella *(j; 30% of development) is crawling out of the shell and marking the end of the metamorphosis.

Developmental stages were recognised by external morphological characters rather than referred to exact points in time after oviposition (po). Typically the embryos of nudibranchs are enclosed by two membranes, the capsule that surrounds each embryo and another mucoid layer that encases all of the capsules in a gelatinous egg mass [41]. After oviposition the first cleavages proceed quickly (at 1-2 hpo two-cell stage (0% of development) and at 8 hpo 16-cell stage (0.5% of development)). The divisions within an egg mass are asynchronous, both four-celled embryos and zygotes can be detected in the same egg mass (Fig. 2A).

Characteristic developmental stages of *Aeolidiella stephanieae*:

#### Early veliger stage (5-10% of development)

The first detectable structures, the larval shell and the ciliated velar lobes, appear at the same time as the first movements of the larvae (rotation around their anterior-posterior axes) (Fig. 2B).

#### Veliger stage (10-20% of development)

The embryo can retract the velum into the shell and the eyes as well as the larval foot (propodium) appear (Fig. 2C).

#### Late veliger stage (20-25% of development)

The operculum is present and the foot becomes thicker and longer (Fig. 2D), the embryo hatches shortly prior to metamorphosis. Swimming is accomplished by ciliary beats of the velar cilia.

#### Metamorphosis (25-30% of development)

Usually one day after hatching the larvae settle on the bottom and retract into the larval shell (Fig. 2E). During the process of metamorphosis, which does not take longer than 48 hours, the animals cast off their larval shell.

#### Early juvenile stage (30-40% of development)

**Figure 3:**
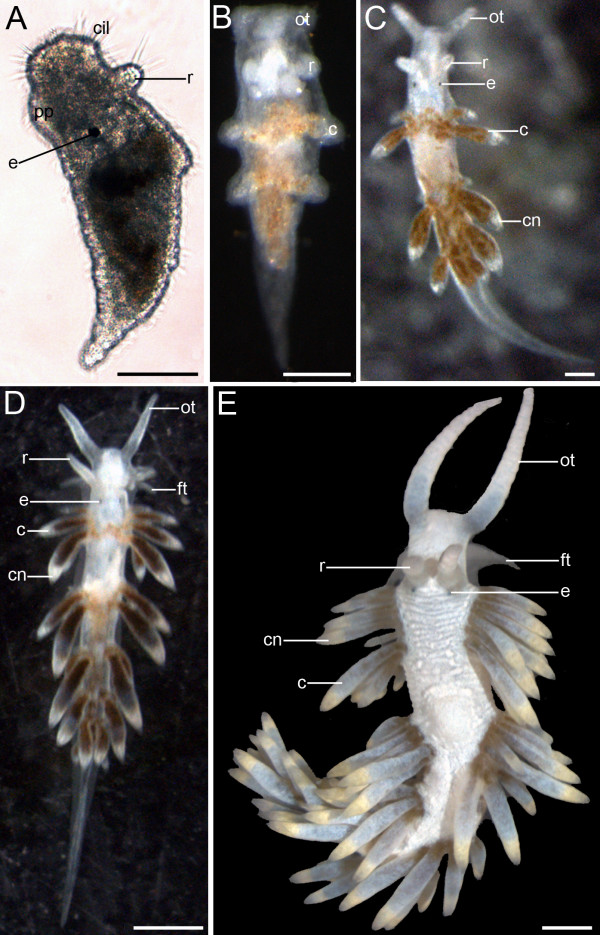
**Postmetamorphic development of *Aeolidiella stephanieae***. Light micrographs. Anterior faces upwards and scale bars represent 100 μm in all aspects. All aspects are in dorsal view except for A which is a lateral view. (A) Early vermiform juvenile *A. stephanieae *(35% of development) with *anlage *of the rhinophores (r). Note that the body is covered by cilia (cil); e marks the eye and pp the propodium. (B) 26 days after oviposition (43% of development) the *anlagen *of the first cerata (c) and oral tentacles (ot) appear. (C) 30 days old juvenile *A. stephanieae *(50% of development) with cnidosacs (cn) at the cerata tips. Note that the rhinophores as well as oral tentacles are longer and thicker now. (D) 39 days after oviposition (65% of development) foot tentacles (ft), a tentacle-like elongation of the propodium, appear. Note that the oral tentacles are almost twice as long as the rhinophores and the additional pairs of cerata. (E) The gross morphology of a mature *A. stephanieae *(100% of development).

Slightly after metamorphosis the early juveniles start to crawl on the bottom, which also marks the beginning of the benthic lifestyle (Fig. 2F). The eyes indicate the anterior part of the white elongated animals. 24 hours after metamorphosis they crawl at the bottom of the culture dish without feeding. At the same time rhinophore rudiments appear anterior to the eyes as the first pair of cephalic tentacles (Fig. 3A). Ciliation of the early juveniles is detectable all over the body (Fig. 3A). At the anterior end and on the tip of the rhinophore rudiments there are cirri, which are compound sensory cilia (Fig. 3A). Generally, 48 hours after metamorphosis juvenile specimens of *A. stephanieae *start to prey upon pieces of *Aiptasia pallida *anemones.

#### Juvenile stage (40-60% of development)

At this stage the rudiments of oral tentacles (2nd pair of cephalic tentacles) and the paired, dorsal cerata appear (Fig. 3B). The size of the body increases one third in contrast to the previous developmental stage. As the development continues, the length and the thickness of the rhinophores and oral tentacles increases as well as the body size (Fig. 3C). At this stage additional pairs of cerata appear and on their tip the filled cnidosacs can be detected for the first time (Fig. 3C).

#### Late juvenile stage (60-90% of development)

As development proceeds, body elongation increases and more pairs of cerata as well as some tentacle-like elongation of the propodium appears (Fig. 3D).

#### First deposition of an egg mass (100% of development)

The gross morphology of the mature *A. stephanieae *is shown in Figure 3E. At this stage the body size is between 0.8-1 cm, which is ten times bigger than in the previous developmental stage, and the oral tentacles are twice as long as the rhinophores. Reproductive maturity is reached 60 dpo (100% of development). The first egg masses are small and contain 60 to 80 embryos. Mature individuals reach a maximum size of 5 cm, and their egg masses contain 1000 to 2000 embryos.

### Neurogenesis of *Aeolidiella stephanieae*

#### Ontogeny of the FMRFamidergic nervous system

**Figure 4:**
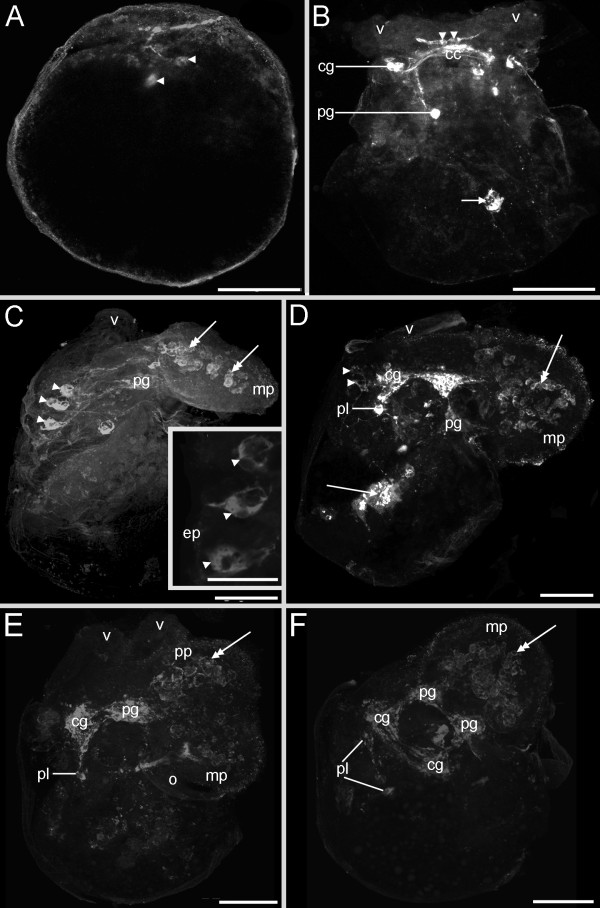
**Confocal micrographs of the FMRFamidergic neurogenesis from the trochophore to the metamorphosis stage of *Aeolidiella stephanieae***. Anterior faces upwards and scale bars represent 50 μm in all aspects expect the inset in C, in which it equals 10 μm. All aspects are in lateral view except for A and B which are in dorsal view. (A) Early veliger larva showing two round cells (arrowheads) of the apical organ in the *anlage *of the velum. (B) Slightly older larva as in A showing cells in the future cerebral (cg) and pedal ganglia (pg), two round apical cells (arrowheads) that lie above the cerebral commissure (cc) and project into the respective lobe of the velum (v), and a FMRFamidergic cell (arrow) in the visceral part of the body. (C) Veliger larva with three sensory cells in the apical organ (arrowheads, inset), the median cell is penetrating the epidermis (ep, inset). Note the FMRFamide positive cells in the larval foot (double-headed arrows); mp marks metapodium. (D) Late veliger larva showing elaborated cerebral, pedal, pleural ganglia (pl), cell clusters in the larval foot (double-headed arrow), and one cell cluster in the visceral body part (arrow). Note the remaining two cells of the degenerating apical organ. (E) Larva at the beginning of the metamorphosis showing the resorption of the velum (v), cells in the cerebral, pedal and pleural ganglia as well as cell clusters in the pro- (pp) and metapodium (double-headed arrow). (F) Larva at the metamorphosis showing the FMRFamidergic nervous system consisting of paired cerebral, pedal, pleural ganglia, and cell clusters in the larval foot (double-headed arrow).

**Figure 5:**
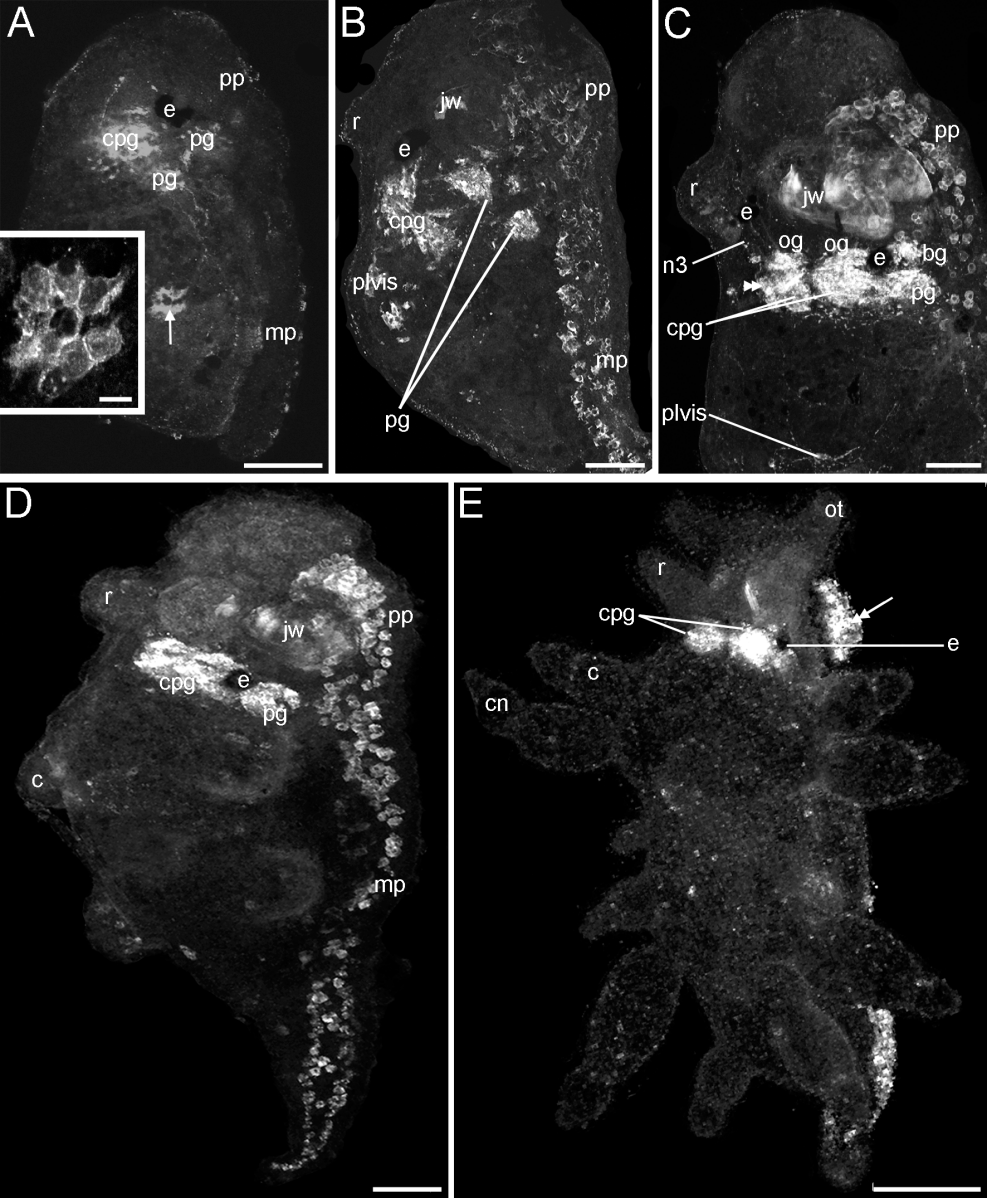
**Confocal micrographs of the postmetamorphic, FMRFamidergic neurogenesis in *Aeolidiella stephanieae***. Anterior faces upwards and scale bars represent 50 μm in all aspects. All aspects are in lateral view. (A) Early juvenile specimen slightly after metamorphosis showing the cerebropleural (cpg) and pedal ganglia (pg). Note the nerve from the pedal ganglion that is running to the cell cluster in the visceral body part (inset, arrow); pp marks the propodium, mp the metapodium, and e the eye. (B) Early juvenile with the *anlage *of the rhinophore (r), jaw (jw) and cerebropleural ganglia, which are connected to the pedal ganglia and the pleurovisceral loop (plvis). Note the FMRFamidergic cells along the entire lengths of the foot. (C) Slightly older early juvenile than in B showing the rhinophoral ganglion (double arrowhead) from which a nerve (n3) is running towards the rhinophore. Note the buccal ganglion (bg) posteroventrally to the jaw, and the optical ganglia laterally to the respective eye (og). (D) Juvenile specimen with the *anlage *of cerata (c) showing the condensed central nervous system where the connective between the cerebropleural ganglion and the pedal ganglion is short. (E) Late juvenile with cnidosacs (cn) on the tips of the cerata and oral tentacles (ot). Note the condensation of FMRFamide positive cells in the anterior part of the foot (double-headed arrow).

The earliest FMRFamide-positive signal is expressed in the early veliger larvae at 5% of development. Two FMRFamidergic cells of the larval apical organ appear synchronously below the early *anlage *of the velum (Fig. 4A). In the early veliger larva at 8% of development slightly posterior to the two apical cells the cerebral commissure is formed (Fig. 4B). As development proceeds some cerebral cells appear on both sides laterally to the apical ganglion, indicating the future cerebral ganglia followed by some FMRFamidergic cells formed dorsoventrally to the ganglia in the *anlagen *of the future pedal ganglia (10% of development, Fig. 4B). At this time an FMRFamide-positive cell appears in the posterior part of the early veliger larva (Fig. 4B). Slightly later (10-15% of development), three cells appear in the apical organ (Fig. 4 inset). The median apical cell is flask-shaped and in contact with the surrounding medium via its neck-like apical projection (Fig. 4 inset). During subsequent development (15-20% of development) the cerebral commissures as well as the cerebropedal connectives become more solid, and more cerebral as well as pedal FMRFamidergic cells emerge (Fig. 4C-D). In addition, more FMRFamide positive cell clusters appear in the larval foot (Fig. 4C-D). At hatching (25% of development) the FMRFamidergic nervous system comprises cerebral-, pleural- and pedal ganglia as well as degenerating apical cells, cell clusters in the larval foot and one cell cluster in the posterior part of the larva (Fig. 4D). When the larvae begin to metamorphose (30% of development), the resorption of the velar lobes starts whereas the FMRFamidergic apical cells already have been resorbed (Fig. 4E-F). The postmetamorphic FMRFamidergic nervous system changes dramatically. The cerebral and pleural ganglia fuse into a single, large cerebropleural ganglion on both sides of the juveniles (Fig. 5A-E). The left and the right cerebropleural ganglia are joined to their respective pedal ganglia via connectives, which are still undergoing the process of fusion (Fig. 5A-D). Early juveniles show a cluster of cells in the posterior part that is innervated from the pedal ganglion (Fig. 5A, inset). The buccal ganglia appear posteroventrally to the autofluorescent jaw and anteroventrally to the cerebropleural ganglia (Fig. 5C). The pleurovisceral loop can be detected posterodorsally to the cerebropleural ganglia (Fig. 5B-C). FMRFamide positive cells are distributed across the foot with a condensation in the anterior part, ventrally to the jaw (Fig. 5B-E). A nerve can be seen exiting each of the rhinophoral ganglion and extending into the developing rhinophores. This nerve is distinguished as the nervus rhinophoralis (n3) (Fig. 5C). The nervus labialis (n2) arising from each cerebropleural ganglion proceeds anteriorly (not shown).

#### Ontogeny of the serotonergic nervous system

**Figure 6:**
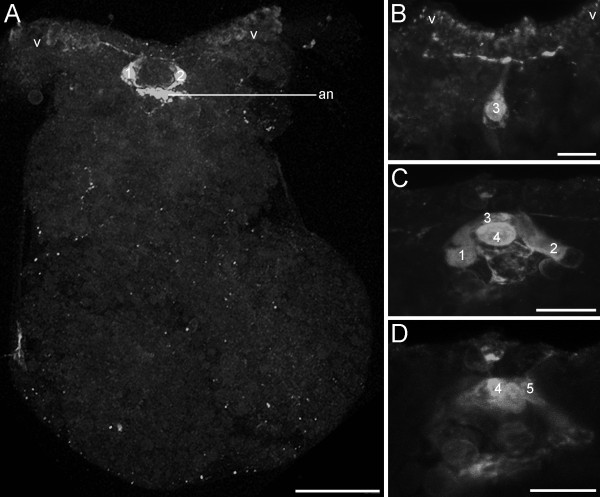
**Confocal micrographs of serotonergic cells in the apical organ of *Aeolidiella stephanieae***. Anterior faces upwards and scale bars represent 10 μm in all aspects expect A, in which it equals 50 μm. All aspects are in dorsal view. (A) Early veliger stage (5% of development) showing two flask-shaped, sensory apical cells (1, 2) above the apical neuropil (an); v marks the velum. (B) An additional median flask-shaped, sensory cell (3) in the apical organ of the early veliger. (C) Veliger larva showing a fourth, round cell in the apical organ (4). (D) Same specimen as in C, showing a fifth, round cell (5) laterally to the fourth cell in the apical organ.

**Figure 7:**
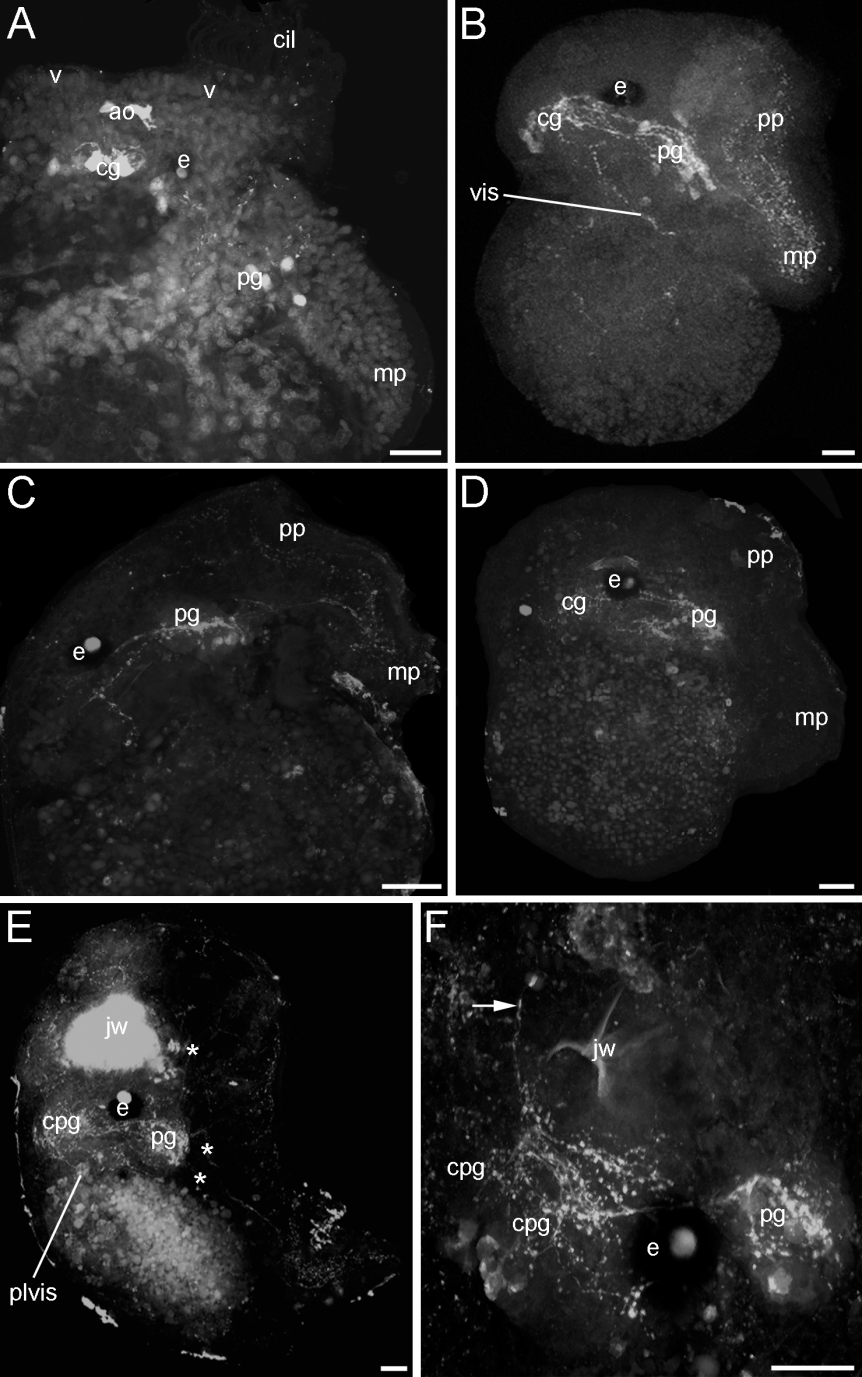
**Confocal micrographs of the serotonergic neurogenesis in *Aeolidiella stephanieae***. Anterior faces upwards and scale bars represent 20 μm in all aspects. All aspects are in lateral view. (A) Veliger larva showing few cells in the pedal (pg) and the cerebral ganglion (cg), which is above the apical organ (ao); cil marks the cilia, e the eye, mp the metapodium, and v the velum. (B) Elaborated nervous system of a slightly later stage as in A with cerebral ganglion joined by several connectives with the pedal ganglion, which innervates the larval foot. Note the visceral loop and the fibres in the larval foot; pp marks propodium. (C) Late veliger larva with additional cells in the pedal ganglia innervating the foot. (D) Larva in metamorphosis showing the cerebral and pedal ganglion. Note that there is no serotonergic positive signal in the apical organ. (E) Early juvenile stage with autofluorescent jaws (jw), the fused cerebropleural ganglion, the pedal ganglion and the pleurovisceral loop. Note the three pedal nerves (asterisk) innervating the foot. (F) Magnification of the anterior head-foot of an early juvenile *Aeolidiella stephanieae *showing cell clusters in the cerebropleural ganglia and a nerve that is running anteriorly (arrow).

Cells containing the neurotransmitter serotonin were labelled in early veliger larvae at 5% of development as early as 3 dpo. Two serotonergic flask-shaped, sensory cells (1 and 2) of the apical organ are located in the anterior region of the larvae (Fig. 6A). In contrast to the FMRFamidergic expression pattern, an apical neuropil is present (Fig 6A). Slightly later, up to five serotonergic apical cells emerge, whereas three of them are flask-shaped, sensory cells (1-3) and two round, non-sensory cells (4 and 5) (Fig. 6B-D). As development proceeds, cerebral and pedal cells appear in the veliger larvae at 10% of development (Fig. 7A). During the following, serotonergic neurogenesis more cells appear in the cerebral and the pedal ganglia (Fig.7B). In addition, fibres of the pedal cells are visible in the larval foot as well as the visceral loop (Fig. 7B). As in the FMRFamidergic expression pattern, no apical cells are detectable closely before and during metamorphosis (Fig. 7C-D). The early juveniles (35% of development) show some serotonergic cell clusters in the cerebropleural and pedal ganglia (Fig. 7E-F). Overall, three distinct serotonergic fibres are running from the pedal ganglia into different parts of the foot (Fig. 7E). In addition, the pleurovisceral loop appears posterior to the cerebropleuralganglia (Fig. 7E). The postmetamorphic condensation shown in the FMRFamidergic expression pattern is also detectable in the serotonergic, indicated by the shortened connectives and commissures (Fig. 7E-F).

#### Pre- and postmetamorphic central nervous system

**Figure 8:**
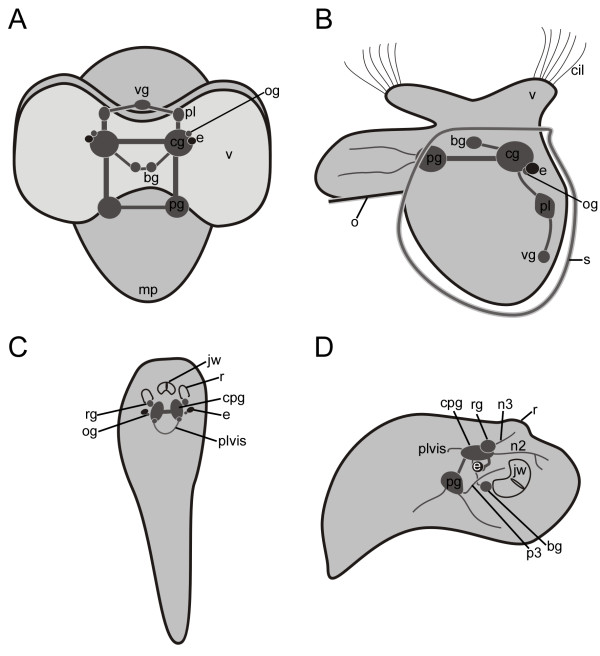
**Schematic representation of the nervous system shortly before (A and B) and after (C and D) metamorphosis in *Aeolidiella stephanieae***. Based on serial thin sections (0,5 μm/section). (A) Frontal view of the nervous system in a metamorphic competent larva (25% of development) showing five paired ganglia; cerebral- (cg), pedal- (pg), pleural- (pl), buccal- (bg), and optical ganglia (og), as well as a unpaired visceral ganglion (vg). The cerebral ganglia are joined via connectives to the pedal-, buccal-, and pleural ganglia. In close proximity to the cerebral ganglia are the eyes (e) as well as their optical ganglia. Commissures are present among cerebral-, buccal-, and pedal ganglia. The connections between the visceral ganglion and the pleural ganglia form the visceral loop; mp marks the metapodium and v the velum. (B) Lateral view of the nervous system shown in A, anterior faces upwards; cil marks the cilia, o the operculum, and s the shell. (C) Dorsal view of the nervous system in an early postmetamorphic juvenile *A. stephanieae *(30% of development), anterior faces upwards. The rhinophoral ganglia (rg) lie at the base of the rhinophores (r) above the newly formed cerebropleural ganglia (cpg). Note that the pleurovisceral loop (plvis) is without a visceral ganglion, which probably fused with one of the cerebropleural ganglia; jw marks the jaw. (D) Lateral view of the nervous system in C, anterior faces right. From the rhinophoral ganglion a nerve (n3) is running to the rhinophore whereas another nerve (n2) is running anteriorly from the cerebropleural ganglion to the lip. Two nerves from the pedal ganglion are innervating the foot, and one is running towards the lip, as well (p3).

The nervous system in late veliger larvae (20-25% of development) in *Aeolidiella stephanieae *consists of five paired ganglia (cerebral-, pedal-, pleural-, buccal-, and optical-ganglia) and one unpaired visceral ganglion (Fig. 8A-B). The buccal ganglia are situated posterior to the mouth and lateral to the oesophagus whereas the cerebral commissure is located above the oesophagus (not shown). The cerebral ganglia, which are the largest ganglia, lie dorsolaterally to the oesophagus and close to the respective eye (Fig. 8A-B). Two nerves are running from the cerebral commissure towards the velar lobes (not shown). At this time, no neural structures of the apical organ are detectable anymore. Located laterally to the eyes and the cerebral ganglia are the small optical ganglia (Fig. 8A-B). The pedal ganglia are situated ventrally to the cerebral ganglia and the pleural ganglia posteriorly (Fig. 8A-B). Both pairs of ganglia, the pedal and pleural, are joined with the cerebral ganglia via connectives (Fig. 8A-B). The pleural ganglia are connected to the posteromedially situated visceral ganglion via connectives, which form the pleurovisceral loop (Fig. 8A-B).

Most evident changes in the anatomy of the nervous system occur during the metamorphosis of *A. stephanieae*. In the postmetamorphic early juvenile *A. stephanieae *(30-40% of development), the cerebral and pleural ganglia fuse to the cerebropleural ganglia (Fig. 8C-D). In addition, only the pleurovisceral loop is still visible but not the single visceral ganglion, which is probably fused with one of the cerebropleural ganglion (Fig. 8C-D). Furthermore, the connectives of all ganglia become shorter, and together with the fusion processes the nervous system becomes condensed. The rhinophoral ganglia, new neural structures, appear laterally to the cerebropleural ganglia (Fig. 8C-D). From each rhinophoral ganglion a nerve is running into the respective developing rhinophore (Fig. 8D). Overall, the nervous system of the early *A. stephanieae *juveniles consists of five pairs of ganglia (cerebropleural-, pedal-, buccal, optical-, and rhinophoral ganglia) (Fig. 8D). The buccal ganglia lie posteriorly to the buccal organ, and the optical ganglia lie near the eyes lateral to the cerebropleural ganglia (Fig. 8C). One nerve is running from the cerebropleural ganglia anteriorly towards the mouth and lip. Two nerves are running from the pedal ganglion, one, the p3-nerve, is running anteriorly towards the lip whereas a second one posteriorly to the mid foot region (Fig. 8D).

### Myogenesis of *Aeolidiella stephanieae*

**Figure 9:**
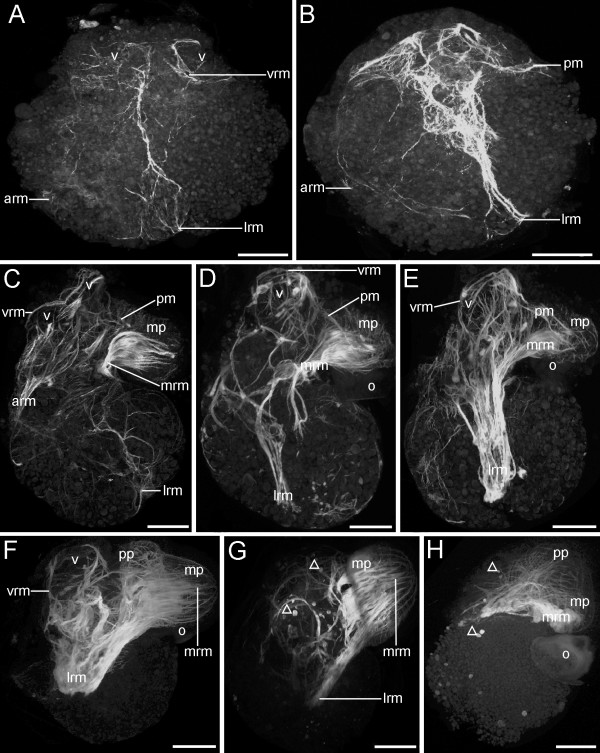
**Confocal micrographs of the myogenesis in premetamorphic and metamorphic developmental stages of *Aeolidiella stephanieae***. Anterior faces upwards and scale bars represent 50 μm in all aspects. All aspects are in lateral view except A which is in dorsal view. (A) Early veliger larva showing first fibres of the larval retractor muscle (lrm), the accessory retractor muscle (arm) and the velar ring muscles (vrm); v marks the velum. (B) Slightly later stage as in A with a more elaborated musculature than in A, and the first fibres of the pedal musculature (pm). (C) The musculature in a veliger consists of retractor muscles, velar ring muscles, pedal muscles, as well as thick metapodial retractor muscles (mrm); mp marks metapodium. (D) Slightly older veliger larva with more elaborate musculature than in C, the retractor muscles are more dominant, the pedal branch of the larval retractor muscle fuses with the metapodial retractor muscle to a thick muscle layer close to the operculum (o). (E) Slightly later stage as in D with a strong larval retractor muscle running to the margin of the velar ring muscle and to the larval foot. (F) In the late veliger stage the most prominent muscle is the larval retractor muscle, and it seems to be fused with the accessory retractor as well as metapodial retractor muscle; pp marks propodium. (G) Larva at the beginning of metamorphosis the velar musculature is degenerating (open triangles). (H) Slightly later stage as in G showing the ongoing degeneration of the larval retractor muscle as well as the velar muscles.

**Figure 10:**
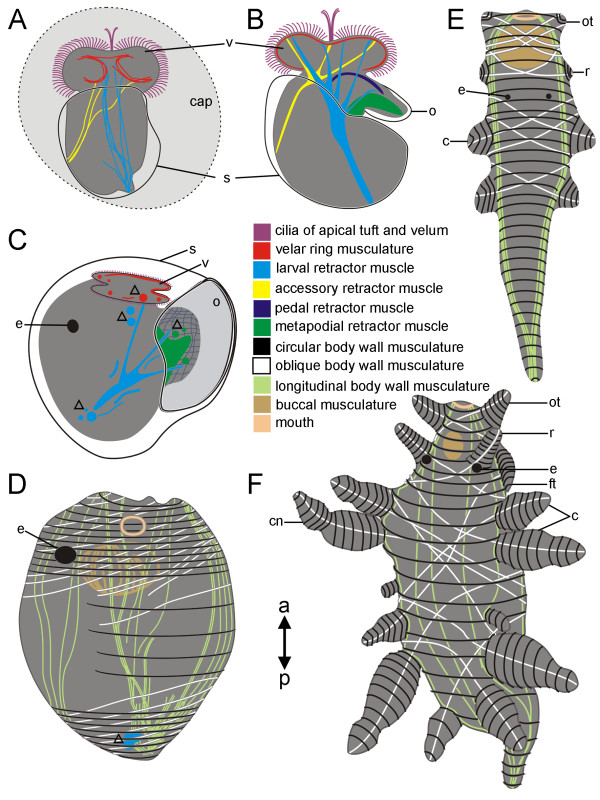
**Schematic representation of myogenesis in *Aeolidiella stephanieae***. Anterior faces upwards and the total size of the specimens is approximately 170 μm in A and B, 120 μm in C, 200 μm in D, 600 μm in E, and 1400 μm in F. "a-p" indicates the anterior-posterior axis of the animals. All aspects are in dorsal view except B, C, and D which are in lateral view. Location of the apical tuft and the velar cilia are indicated (purple) as well as the mouth opening (rosy). (A) Early veliger stage larva (5% of development) showing first fibres of the larval retractor muscle (cyan), the accessory retractor muscle (yellow) and the velar ring muscles (red); v marks the velum, s the shell, and cap the capsule. (B) Veliger stage larva (10% of development) with well developed accessory, larval, pedal (blue), and a metapodial (green) retractor muscle; o marks the operculum. (C) Into the shell retracted metamorphic larva (25% of development) showing degenerating velar lobes and muscles (open triangles); e marks the eye. (D) Closely after metamorphosis (30% of development) the body wall musculature comprises longitudinal (light green), circular (black), and oblique (white) muscle fibres. Note the *anlage *of the buccal musculature (brown) and that the larval retractor muscle is still degenerating. (E) Juvenile *Aeolidiella stephanieae *(40% of development) with the *anlage *of oral tentacles (ot), rhinophores (r), and the first cerata pairs (c). (F) Late juvenile *A. stephanieae *(65% of development) showing the meshwork of outer circular, intermediate oblique, and inner longitudinal body wall muscle fibres as well as growing tentacles and the cerata with cnidosacs (cn); ft marks foot tentacle, a tentacle-like elongation of the propodium.

**Figure 11:**
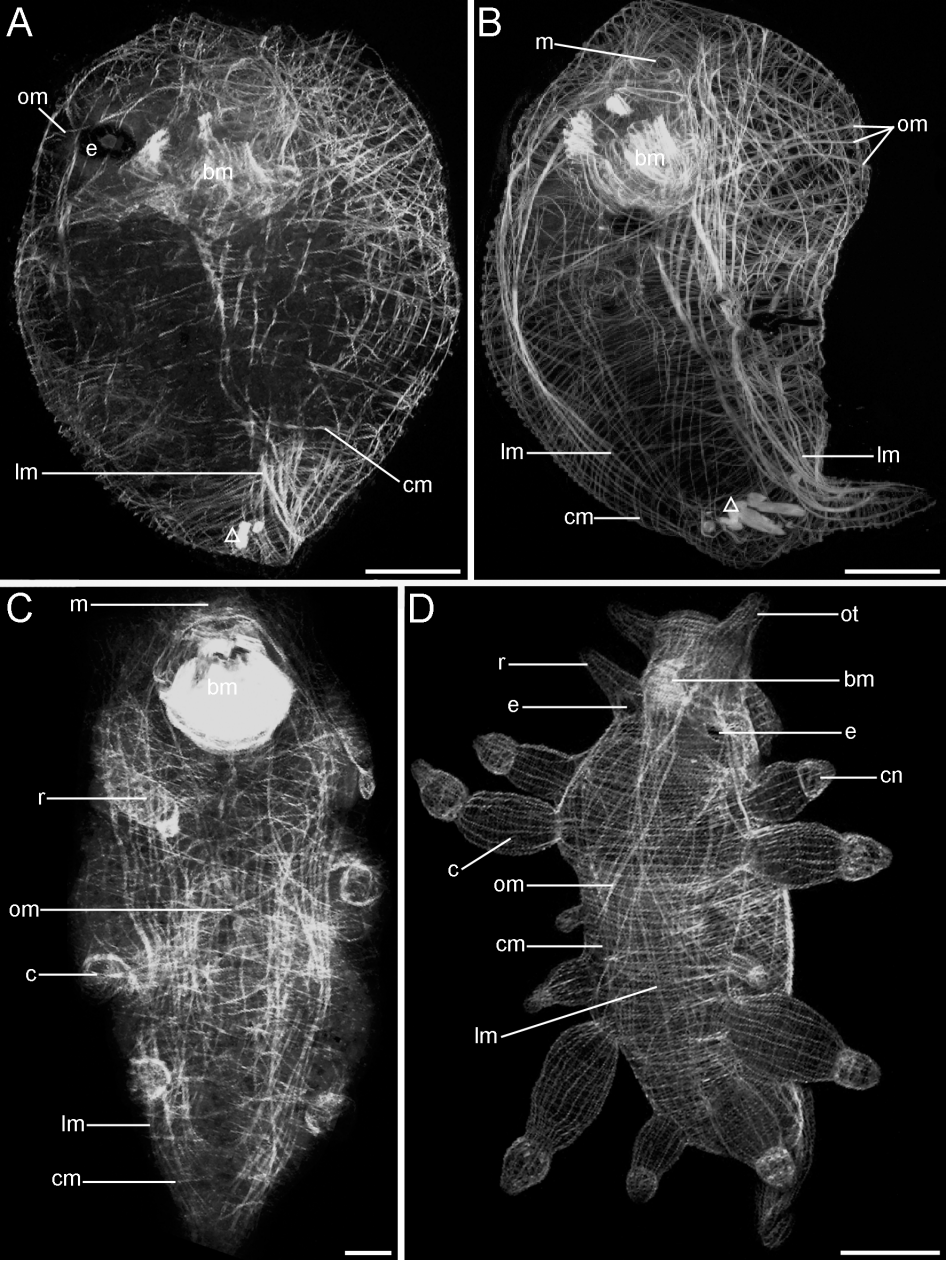
**Confocal micrographs of the myogenesis in postmetamorphic developmental stages of *Aeolidiella stephanieae***. Anterior faces upwards and scale bars represent 50 μm in all aspects expect D, in which it equals 200 μm. All aspects are in lateral view except C and D which are in dorsal view. (A) Early juvenile *Aeolidiella stephanieae *closely after metamorphosis showing the body wall musculature comprising longitudinal (lm), circular (cm) and oblique muscle fibres (om). Note that the larval retractor muscle is still degenerating (open triangle); bm marks the buccal musculature and e the eye. (B) Early juvenile specimen with outer circular, inner longitudinal and intermediate oblique muscles; m marks the mouth. (C) Juvenile stage with cerata (c) and rhinophores (r). (D) The musculature of the growing tentacles and cerata in a late juvenile *A. stephanieae *comprises longitudinal and transverse muscle fibres. The body wall consists of a tight meshwork of circular, longitudinal and oblique muscle fibres; cn marks the cnidosac and ot the oral tentacle.

At the early veliger stage (5% of development) fibres of the larval retractor muscle run from the insertion area, located on the dorsoposterior internal protoconch, slightly to the left side with respect to the medial plane, to the *anlage *of the velar musculature (Figs. 9A and 10A). At the same time the accessory larval retractor appears dorsolaterally to the larval retractor muscle and runs anteriorly to the *anlage *of the velum (Figs. 9A and 10A). The velar branch of the larval retractor muscle comprises two muscle bundles with fibres running into the velar lobes and terminating at the velar margin, the velar ring muscle (Figs. 9A and 10A). When myogenesis continues, the larval retractor muscle and the accessory retractor muscle become more solid as well as the anterior musculature, comprising the velar lobes and the pedal musculature (Fig. 9B). At 10% of development the veliger larvae exhibit a well differentiated pro- and metapodial musculature, comprising longitudinal fibres, pedal muscles and metapodial retractor muscles (Figs. 9C and 10B). At the same time the velar musculature is well developed, comprising a velar ring muscle and longitudinal muscle fibres, which run from the larval retractor muscle and the accessory retractor muscle to the velar ring muscle (Figs. 9C and 10B). In the following development the larval retractor muscle becomes a dominant retractor muscle (Fig. 9D-F). The larval retractor muscle comprises a muscle branch that extends into the velum and another one running into the pedal region (Fig. 9D-F). The broad thick muscle layer situated in close proximity to the operculum is a fusion of the pedal branch of the larval retractor muscle and the metapodial retractor muscle (Fig. 9D-G). The pedal muscle anatomy is composed of a set of different muscles. It starts in the early veliger stage (5-10% of development), where some longitudinal fibres run from the velum into the *anlage *of the future larval foot (Fig. 9B), and continues with the establishment of the metapodial retractor muscle and a net of longitudinal and transversal muscle fibres in the veliger (10-20% of development) and metamorphic (25-30% of development) developmental stages (Fig. 9C-H). Slightly after hatching at 20-25% of development the larvae exhibit intensely fluorescing dots near to the velar musculature and the larval retractor muscle (see open triangles in Figs. 9G-H and 10C). These dots most likely represent depolymerised F-actin indicating velar and larval retractor muscle degeneration during metamorphosis. In fact, all larval muscles (the larval retractor muscle, the accessory larval retractor muscle, the velar muscles, and the pedal retractors) degenerate throughout metamorphosis or slightly after, and the post-metamorphic myo-anatomy is formed *de novo*. At 30% of development a new structure, the buccal musculature, appears after metamorphosis (Figs. 10D and 11A). The larval retractor muscle remains indicated by intensely fluorescing structures in the posterior end of the early juvenile, and is still in the process of degeneration (Figs. 10D and 11A-B). The complex post metamorphic myo-anatomy of the early juvenile comprises circular body wall muscles as well as longitudinal and oblique muscles (Figs. 10D and 11A-B). In the following myogenesis the buccal musculature migrates anteriorly and is located at the anterior end between the eyes (Figs. 10D-F and 11B-D). Longitudinal muscle fibres accumulate to ventrolateral muscle strands, and run from the anterior to the posterior end of *Aeolidiella stephanieae *juveniles (Figs. 10D-F and 11B-C). Several oblique muscle fibres that originate at the ventral anterior side of the juvenile *A. stephanieae *loop in a dorsal direction, and eventually bend again towards the ventral side (Fig. 11B-D). The growing tentacles and cerata are formed by a tight meshwork of longitudinal and transversal muscle fibres (Fig. 11D).

## Discussion

### General aspects of developmental modes in Mollusca

Briefly, there are three main types of opisthobranchiate development: one where a planktotrophic larval stage is obligatory, one where development is via a free swimming lecithotrophic larval stage, and one where the intracapsular development ends post metamorphosis (direct development) [42,43]. Since the lecithotrophic life history pattern is present in basal mollusc (e.g. Solenogastres, Caudofoveata and, Polyplacophora) and basal gastropod groups (e.g. Patellogastropoda, Vetigastropoda), there is growing consensus that the lecithotrophic development is ancestral for molluscs [36,44-49]. In nudibranchs planctotrophic [50], lecitothrophic [51] and direct development [43] are present. All three developmental strategies can be found in closely related taxa and genera. Hence, the life history pattern in Nudibranchia does not reflect the systematic position of a taxon or species.

In *Aeolidiella stephanieae *the development is lecithotrophic. The first pair of cephalic tentacles, the rhinophores, emerge shortly after metamorphosis (30% of development), whereas the second pair, the oral tentacles, appear significantly later in postmetamorphic stages (juvenile stage, 40% of development). The same developmental pattern of cephalic tentacles has been shown in three other nudibranchs, so far (*Adalaria proxim*a [52], *Cadlina laevis *[43], and *Melibe leonina *[50]). In contrast, the rhinophores of the nudibranch, *Rostanga pulchra*, emerge shortly before metamorphosis [53], and in the aplysiomorph *Aplysia californica *the oral tentacles emerge first and the rhinophores later in development [54,55]. The cephalic tentacles are important sensory organs that function as chemo-, photo-, and mechanoreceptors [56-59]. Because of these organs, gastropods are capable of locating and discriminating food, predators, and conspecifics [60-63], and orienting themselves toward water currents [64,65]. Oral tentacles of adult opisthobranchs function as contact chemoreceptors [59,66,67] to taste food quality, whereas rhinophores are distant chemoreceptive sensory organs used to locate distant food sources [59,61,68]. Since *A. californica *larvae need to settle before metamorphosis on their future diet, red seaweed, they have to be able to taste the food quality first and do not need rhinophores, as yet [54]. Seemingly, settlement and metamorphosis in *A. stephanieae *larvae are not triggered by their future prey, and most likely therefore the rhinophores develop first after metamorphosis in order to be able to locate their diet, sea anemones. This implies that the developmental sequence of cephalic tentacles is an adaptation to the way of living rather than a character reflecting phylogenetic relationships in different opisthobranch taxa.

### Phylogenetic significance of neuro- and myogenesis in gastropods

Immunocytochemical studies of nervous system and F-actin labelling of musculature of gastropods from early larval to postmetamorphic stages are rare [2,24,55]. In addition, many other studies deal only with selected developmental stages (e.g. preveliger, hatchlings, and metamorphic larvae) [69-72]. Our immunocytochemical data increase the knowledge of neuro-and myogenesis and thus enable the comparison of certain features of opisthobranch and gastropod development, which is significant in view of their phylogenetic history.

#### Apical organ

**Figure 12:**
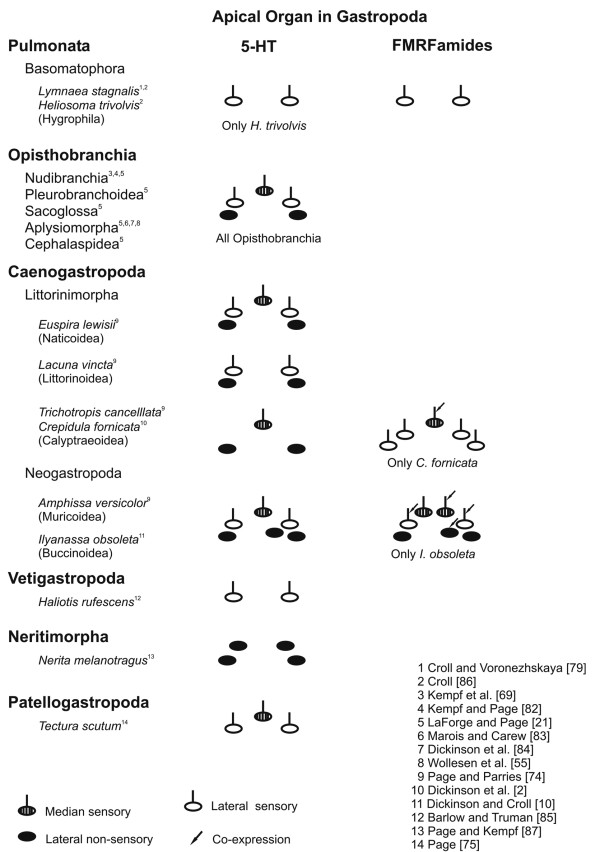
**Arrangement of serotonergic and FMRFamidergic positive cells within the apical organ in gastropod larvae (redrawn and modified from Page and Parries **[74]**)**. Anterior faces upwards and the symbols on the bottom identify the three categories of neurons within the apical organ. The arrow identifies cells within the apical organ that co-express serotonin and FMRFamide. Classification after Ponder and Lindberg [47].

The best studied cells in the apical organ of gastropods have been those immunoreactive to serotonin (monoamine) (Fig. 12) [10,21,55,69,73-75]. Other neuronal markers such as catecholamines [71,76,77] and neuropeptides including FMRFamide [78-81] have also been identified within apical cells in various gastropod species. The types and numbers of apical cells containing catecholamines and FMRFamide show fewer similarities than do those cells containing serotonin. Five apical cells are immunoreactive for FMRFamide and the same number for serotonin during the early veliger and veliger stage (5-15% of development) of *Aeolidiella stephanieae*. Two flask-shaped sensory cells flank a median flask-shaped cell with two round/paraampullary cells beneath, all, situated above the cerebral commissure. Although the five FMRFamidergic and serotonergic apical cells show a similar arrangement, since no double labelling experiments have been performed we cannot say whether they are identical or not. However, in the caenogastropods *Crepicula fornicata *[2] and *Ilyanassa obsoleta *[10] various apical cells contain both the monoamines and the neuropeptides. Further experiments will clarify, whether this is also true for some or even all of the immunoreactive apical cells of *A. stephanieae*. Besides the co-expression of one or more neurosubstances, we note that the caenogastropod *Euspira lewisii *(Littorinimorpha, Naticoidea) [74] and all opisthobranchs studied to date have identical arranged types of five serotonergic neurons within the apical ganglion [21,55,69,73,82-84]. They all show three sensory and two non-sensory serotonergic cells within the apical organ. As indicated in the comparative sketches in Figure 12, caenogastropods are particularly notable for interspecific differences in the number of serotonergic cells within the apical organ. Littorinimorph caenogastropods that belong to the clade of Littorinoidea, for example, lack the median sensory cell, the ones of calyptraeoidean clade lack the lateral sensory cells instead, whereas all investigated neogastropod clades have an additional lateral non-sensory, round cell [2,10,74]. It has been suggested previously that the presence of the unpaired lateral cell in the neogastropods *I. obsoleta *and *Amphissa versicolor *might be related to one larger velar lobe and/or the earlier development of the rhinophores, which are already present slightly before the metamorphosis [6]. Congruent with this assumption is that the lecitothrophic (non-feeding) patello- and vetigastropod larvae, which have a smaller velum, have only three and two serotonergic apical cells, respectively [75,85]. In addition, there are only two serotonergic apical cells in the direct developing embryos of pulmonates [79,80,86]. Neritimorpha, Opisthobranchia and Caenogastropoda are all groups that have feeding larvae (planktotrophic development) and an increase in serotonergic cells within the apical organ (Fig. 12). Moreover, the planktotrophic life history pattern with a long-term, planktotrophic larva is assumed to be ancestral for these three gastropod lineages [21,74,87,88]. The question whether larval planktotrophy arose independently in these lineages or whether it was shared with a common ancestor is still under discussion [47,74,87-90]. However, Page and Kempf [87] described significant differences in the structure of the apical organ between the larvae of Neritimorpha and Apogastropoda (Caenogastropoda + Heterobranchia), and therefore suggested an independent origin of planktotrophy in these two gastropod groups. In fact, the apical organ of caenogastropod and opisthobranch larvae is strikingly similar [21,74]. In addition, Apogastropoda has been recognized independently in morphological, molecular and combined analyses as the most derived clade whereas Patellogastropoda and Vetigastropoda are members of the more basal clades (Fig. 1A-B). This correlates with the fact that the patello- and vetigastropods have only few serotonergic cells in their apical organ and lecithotrophic development, which is assumed to be the plesiomorphic life history pattern for the gastropods as a whole [47,91]. Hence, it is doubtful that Caenogastropoda is the most basal clade within Gastropoda as recently suggested by mitochondrial genome arrangements (Fig. 1C).

Regardless of the differences in serotonergic cell numbers, several studies support the assumption that the apical organ plays an important role in metamorphic processes like settlement [see [6,72,92,93]]. However, loss of serotonergic and FMRFamidergic cells in the apical organ in several gastropods and the scaphopod *Antalis entalis *well before the onset of metamorphosis challenges this assumption [5,55,94]. Accordingly, the role in settlement and metamorphosis might not be the ancestral function of the apical organ. No FMRFamidergic or serotonergic signal can be detected in the apical organ of *Aeolidiella stephanieae *larvae shortly before or throughout the metamorphosis (20-30% of development). Degeneration of the apical organ throughout metamorphosis has also been shown in another opisthobranch gastropod, *Aplysia californica *[55,83]. In the caenogastropod *Ilyanassa obsoleta*, loss of cells in the apical organ has been demonstrated to occur during metamorphosis through some form of programmed cell death [94]. It has also been shown that some apical cells of *I. obsoleta *can be respecified and migrate into adjacent ganglia [10]. Nevertheless, a general feature of gastropod neurogenesis is seemingly that the larval nervous system (apical organ, the serotonergic velar/prototroch nerve ring) is lost before, during, or shortly after metamorphosis [49].

#### Central nervous system and periphery

The neurogenesis of *Aeolidiella stephanieae *is similar to that of other nudibranchs [21,70,95]. The larval nervous system of *A. stephanieae *includes an apical organ, developing central ganglia, and peripheral neurons associated with the velum, foot and posterior part of the larvae. The first neurons containing serotonin and FMRFamide are observed during the early veliger stage (5-10% of development) in the apical organ. Slightly later, in the veliger stage (15% of development), peripheral FMRFamidergic cells appear in the posterior part of the larvae, and persist throughout metamorphosis into the early juvenile stage (30% of development). In other gastropods, these neurons have never been documented to persist during metamorphosis. Similar posterior neurons containing FMRFamide were first described in the pulmonate *Lymnaea stagnalis *at a time analogous to the trochophore stage [78,79]. Since then they have also been described in trochophore stages of the opisthobranch *Aplysia californica *[84], as well as the caenogastropods *Crepidula fornicata *[2] and *Ilyanassa obsoleta *[10].

As in many other gastropods, the ganglia of *Aeolidiella stephanieae *develop from an anterior to posterior direction in both expression patterns, serotonergic and FMRFamidergic, where the cerebral ganglia develop first followed by the pedal-, and the posterior ganglia [55,96]. In contrast, there are also reports where some of the earliest serotonergic or FMRFamidergic cells appear well before the cerebral and pedal ganglia in the more posterior osphradial, intestinal, abdominal, and parietal ganglia, respectively [2,10,79,84,92]. These findings contradict the prior suggestion that gangliogenesis in gastropods develop in an anterior to posterior sequence [97-99]. However, single serotonergic and FMRFamidergic cells as well as fine neurites can be visualised by immunolabellings, but it does not reflect the entire nervous system. Light microscopy and serial sections, in contrast, might show almost all parts of the nervous system but not the very fine neural structures. Therefore, a comparative combined immunocytochemical, histochemical and TEM analysis might deepen the knowledge in gastropod and mollusc gangliogenesis.

Regardless of which ganglia develop first, the central ganglia that develop during the larval stages will persist the metamorphosis and become the adult CNS in gastropods and other molluscs (reviewed in Croll and Dickinson [6]).

As in other nudibranchs described, the CNS of *Aeolidiella stephanieae *becomes more concentrated during metamorphosis [51,95,100-103]. In *Berghia verrucicornis *the pleural and cerebral ganglia fuse together to cerebropleural ganglia, the visceral ganglion fuses with the left cerebropleural ganglion to become the pleurovisceral loop, and all the connectives become shorter [95]. This is congruent with our findings in *A. stephanieae *except that we can not document to which of the newly formed cerebropleural ganglia the visceral ganglion becomes part of. However, in the newly metamorphosed *A. stephanieae *rhinophoral ganglia appear as additional neural structures at the same time as the rhinophores start to grow. This is also true for the nudibranchs *Adalaria proxima*, *Tritonia hombergi*, *Cadlina laevis*, *Rostanga pulchra*, *Melibe leonina*, and *B. verrucicornis *[43,52,53,95,100,102,103]. In contrast, in the aplysiomorph *Aplysia californica *all connectives become longer, and the ganglia do not fuse [55,104]. Furthermore, in *A. californica *the oral tentacles and their ganglia appear well before the rhinophores and their respective neural structures [55,104]. Since Nudibranchia is considered a derived taxon compared to Aplysiomorpha [29,30,105,106], the condensation of the CNS during metamorphosis and the absence of oral tentacle ganglia thus appear to be a derived condition of certain opisthobranch clades.

#### Myogenesis

F-actin labelling in conjunction with cLSM enables precise reconstruction and visualisation of three dimensionally arranged muscles systems of microscopic invertebrates [107-111]. So far, three "basal" [112-114] and one "higher" gastropod [24] have been investigated applying this technique. The primitive gastropods (patello- and vetigastropods) have four larval muscle systems (main and accessory larval retractors, velar and pedal muscle system) [113-115]. The larval retractors and the velar musculature degenerate during or shortly after metamorphosis, while the pedal muscle plexus continues into adult musculature, and the buccal, tentacle, and shell musculature originate independently from all other muscles [114,115]. In contrast, existing studies on caenogastropod larvae consistently describe only one shell attached retractor muscle and one pedal muscle [42,116-123]. The larval retractor muscle makes no contribution to the post-metamorphic adult shell muscle (columellar muscle) because it derives from a portion of the larval pedal muscle only [123]. However, although only four taxa of opisthobranchs have been investigated so far, they are particularly notable for variations in number and configuration of larval shell muscles (Cephalaspidea [124,125], Aplysiomorpha [24,126], Sacoglossa [127,128], Nudibranchia [51,100,129-134]). Cephalaspidea and Aplysiomorpha have the main and the accessory larval retractor muscles, [24,124-126] whereas Sacoglossa and the anthobranch Nudibranchia lack the accessory larval retractor muscle [51,100,127-129,131,132,134]. Casteel [130] showed three retractors for *Fiona marina*, while Bonar and Hadfield [133] reported only two in *Phestilla sibogae *(both cladobranch nudibranchs). This is congruent with our observations in *Aeolidiella stephanieae *where the accessory larval retractor muscle is present. In other opisthobranchs than nudibranchs, the cephalopedal musculature contributes to the adult muscle complex. As in other nudibranchs the post-metamorphic myo-anatomy in *A. stephanieae *is formed *de novo*. However, regardless the number, larval retractor muscles make no contribution to the post-metamorphic columellar muscle in opisthobranchs. Interestingly, in Patello-, Veti-, Caenogastropoda, and Opisthobranchia the buccal and tentacle musculature originates independently from all other muscles, and, with the exception in nudibranch gastropods, the cephalopedal musculature plays an important role in differentiation of adult musculature. Therefore, the *de novo *formation of the adult muscle complex is probably a derived nudibranch character. Moreover, ultrastructural studies on patellogastropods, nudibranchs, and the caenogastropod *Polinices lewisii *showed that the larval and, if present, the accessory retractor muscle is obliquely striped [114,115,123]. Accordingly, *A. stephanieae *bears similarities with other gastropods in terms of target tissues (velar/prototrochal and cephalic regions) and their developmental fate, i.e., the degeneration of the larval retractor muscles before or during metamorphosis. Furthermore, as also found in other gastropods, *A. stephanieae *exhibits a larval retractor muscle that is anchored on the posterior inner shell and inserts at a muscle ring that underlies the prototroch or velum, which enables retraction of the velum into the mantel cavity (Opisthobranchia [24,52,124,126,133,134], other Gastropoda [112-115,135]). Accordingly, it is likely that the retractor muscles in Gastropoda are homologues, and therefore the patello- and vetigastropod condition with an accessory and a larval obliquely striped retractor muscle plesiomorphic. Hence, the caenogastropod and the two opisthobranch groups, Sacoglossa and Anthobranchia with only one larval retractor muscle represent a derived condition. This is congruent with several morphological and molecular phylogenetic analyses where the sister taxon relationship are found between Cephalaspidea and Aplysiomorpha as well as Anthobranchia and Cladobranchia [24,29,30,105,106,136]. Moreover, several analyses indicate that the Sacoglossa might have a closer relationship to the Nudibranchia (Anthobranchia + Cladobranchia) [27,30,137].

### Evolutionary derivation of neuromuscular patterns of gastropods compared to other molluscs

Within molluscs there are variations in the number of apical cells. Four ampullary serotonergic cells have been reported in the apical organ of scaphopods [5], as well as eight to ten ampullary and two para-ampullary serotonergic cells in polyplacophorans [3,138]. Recently, the bivalve *Mytilus trossulus *was shown to possess five FMRFamidergic and serotonergic apical sensory cells [8], although again, these numbers vary among bivalves [139,140]. The close temporal and spatial association of the apical organ with the developmental onset of the cerebral commissure is a general feature of neurogenesis in molluscs. This supports the assumption that the larval apical organ plays an inductive role in the formation of the future adult cerebral nervous system. This corresponds to the situation in annelid and sipunculan larvae investigated so far, and might represent a plesiomorphic condition of trochozoan neurogenesis [20,22,141-143].

Recently, in the bivalve *Mytilus trossulus *three pairs of larval striated retractor muscles that degenerate throughout metamorphosis were described [144]. Furthermore, bivalve larval retractor muscles insert at a muscle ring that underlies the prototroch or velum as also found in *Aeolidiella stephanieae *and other gastropods [144,145]. In contrast, scaphopods lack most of the larval retractor muscles as well as a prototroch muscle ring, and polyplacophoran larvae do not exhibit a retractor muscle but a prototroch muscle ring [146,147]. The fact that the prototrochal muscle ring is found in Polyplacophora, Bivalvia, and all Gastropoda with a larval stage, but not in Scaphopoda suggests a secondary loss in scaphopods. However, a prototroch muscle ring is present in other trochozoans such as polychaetes [49] and sipunculans [148] indicating that this structure might represent a plesiomorphic character for Mollusca. In addition, the strikingly similar larval retractor muscle between gastropods and bivalves might reflect their close phylogenetic relationship [149]. The prototrochal muscle ring as well as a body wall musculature comprising outer ring, intermediate oblique and inner longitudinal muscles, is, in contrast, a shared feature among trochozoans [25]. In nudibranch gastropods, however, this is a secondary condition since the majority, including the basal gastropod groups (i.e. Patello- and Vetigastropoda) do not have this kind of body wall musculature.

## Conclusion

The similarity of the structure and arrangement of neurons in the apical organ expressing serotonin-like immunoreactivity between Caenogastropoda and Opisthobranchia indicates their sister taxon relationship as shown by previous morphological, molecular and combined analyses (Fig. 1A-B). Consequently, the accessory larval retractor muscle of the basal gastropods, Patello-, and Vetigastropoda, and *Aeolidiella stephanieae *is part of the gastropod ground pattern that has been lost in Caenogastropoda, Sacoglossa and anthobranch nudibranchs. Accordingly, the placement of Caenogastropoda as the most basal gastropod clade as currently suggested by Grande et al. [39]; Fig. 1C] is not supported.

## Methods

### Animal Culture

Adults of *Aeolidiella stephanieae *Valdéz, 2005 and several specimens of *Aiptasia pallida *(Cnidaria, Anthozoa) were purchased from pro-marin of the Justus-Liebig-University in Giessen (Hessen/Germany). Both the sea anemones and the gastropod molluscs were cultured at the University of Frankfurt am Main (Hessen/Germany). The sea anemone, *A. pallida*, (used to feed *A. stephanieae*) was reared in a 200 litre tank with artificial seawater at 18°C temperature and fed with nauplius larvae of *Artemia salina *(Crustacea, Brachiopoda) every two to three days. Other, more detailed, methods for culture of *A. pallida *have been described by Hessinger and Hessinger [150].

Adult specimens of *Aeolidiella stephanieae *were kept in a 20 litre tank at a water temperature of 21°C filled with approximately 5 litre of 0.45 μm millipore-filtered artificial seawater. The metabolic and digestive wastes of invertebrate species and algae from this aquaria provide for the growth of essential bacterial populations that desoxify ammonia and nitrites. The water was changed once weekly. Freshly laid egg masses were transferred to unaerated 60 ml glass crystallizing dishes (VWR, Darmstadt, Germany, ø 60 mm, volume 60 ml, height 35 ml) containing 30-50 ml of 0.45 μm millipore-filtered aquarium water of 22°C. One or two small *Aiptasia pallida *were placed into every second dish, or pieces of the foot were placed in the dish together with the *A. stephanieae *two days after metamorphosis. Thereafter, sea anemones were added and the water was changed every second day. As the specimens reached the last juvenile stage (fully developed rhinophores, labial tentacles and cerata) they were transferred in unaerated 500 ml glass crystallizing dishes containing 300-350 ml of 0.45 μm millipore-filtered aquarium water. At the time a specimen laid the first egg mass it was considered to be sexually mature. Thereafter the specimens were added to the adults in the 20 litre aquaria. Antibiotics were not used in any stage of culture. Regular water changes were sufficient to prevent high infestation with protist and bacterial contaminants.

### Developmental staging

The development of *Aeolidiella stephanieae *lasts 60 days at 22°C based on morphological, morphometrical and behavioural features examined at regular time intervals throughout development. Stages are expressed as a percentage of development, wherein 0% corresponds to the first egg cleavage and 100% to the first layed egg mass (error margin of 2%). The results are based upon observations of at least 300 specimens at each of the premetamorphic, metamorphic and early postmetamorphic (30-40% of development) developmental stages and at least 30 specimens at each of the later, juvenile developmental stages.

### Immunolabelling and confocal laser scanning microscopy

Larvae were anesthetised by adding drops of 7% MgCl_2 _solution to seawater and were subsequently fixed in 4% paraformaldehyde (PFA) in 0.1 M phosphate-buffered saline (PBS) (pH 7.3) for 4-6 h at 4°C. This procedure was followed by rinsing the specimens in 0.1 M PBS (pH 7.3) three times (5, 5 and 60 min). Larvae were decalcified with 0.5 M ethylenediaminetetraacetic acid (EDTA) in aqua dest. for several hours. Afterwards, the animals were rinsed in 0.1 M PBS (5, 5, 60 min), permeabilised (4% Triton X-100 in PBS 0.1 M for 24 h) and incubated in one of the antibodies listed below in a blocking solution (1% normal goat serum in PBT (0.1 M PBS containing 1% Triton X-100)) for 72 h at 4°C. Both antibodies were diluted in PBS (0.1 M) and applied at a 1:250 (rabbit anti-Serotonin; Acris, Hiddenhausen, Germany) or a 1:500-1:1000 (rabbit anti-FMRFamide; Immunostar, Hudson, Wisconsin, USA) final working concentration. After several washes in 0.1 M PBS, a goat anti-rabbit secondary antibody conjugated to fluorescein isothiocynate (FITC) (Jackson ImmunoResearch Laboratories, Inc., West Grove, USA) or rhodamine (TRITC) (Jackson ImmunoResearch Laboratories, Inc., West Grove, USA) was applied at a 1:50 dilution in 0.1 M PBS for 24 h at 4°C, which was then followed by several washes in PBS. All specimens prepared for immunocytochemistry were mounted in a 3:1 mixture of glycerol to TRIS-buffer (0.5 M) with 2% propyl gallate added to prevent fading [151] on glass slides. As negative controls, animals were processed without incubation in primary antibody resulting in no fluorescence labelling. Positive controls included parallel processing of adult central nervous system of *Littorina littorea *and *Haminoea japonica *with known labelling patterns (Staubach (Field Museum Chicago) and Schulze (Zoological Museum Hamburg) pers. comm.).

For F-actin labelling, anesthetised and fixed (see above) larvae were washed in 0.1 M PBS (3 × 15 min. at room temperature), permeabilised for 6 h PBT at 4°C, and incubated in a 1:40 dilution of the fluorescent dye Oregon Green 514 phalloidin (Molecular Probes, Eugene, Oregon, USA) in 0.1 M PBS in the dark for 1 h at room temperature. Afterwards, the samples were washed again (3 × 15 min) and mounted (see above) on glass slides.

Analysis and digital image acquisition of the fluorescence preparations was performed on a Leica DM LB microscope and a Leica TCS SP5 confocal laser scanning microscope. Optical sections taken at intervals of 0.1-0.5 μm were generated and digitally merged to maximum projections. Images were further processed with Photoshop 6.0 (Adobe Systems, San Jose, California, USA) to adjust contrast and brightness. In addition, drawings were created with the computer based software Corel Draw 11.0 (Corel Corporation, Ottawa, Ontario, Canada).

## Competing interests

The authors declare that they have no competing interests.

## Authors' contributions

AKK designed and coordinated the study. AK performed the investigations, analysed the data and drafted the manuscript. AKK commented on earlier versions of the manuscript. Both authors read and approved the final manuscript.
